# A Review on Rhubarb-Derived Substances as Modulators of Cardiovascular Risk Factors—A Special Emphasis on Anti-Obesity Action

**DOI:** 10.3390/nu14102053

**Published:** 2022-05-13

**Authors:** Oleksandra Liudvytska, Joanna Kolodziejczyk-Czepas

**Affiliations:** Department of General Biochemistry, Faculty of Biology and Environmental Protection, University of Lodz, 90-236 Lodz, Poland; joanna.kolodziejczyk@biol.uni.lodz.pl

**Keywords:** anti-lipidemic action, obesity, rhubarb, rhubarb fibre, adipocyte, adipogenesis

## Abstract

The currently available anti-obesity therapies encounter many associated risks and side effects often causing the ineffectiveness of treatment. Therefore, various plant-derived substances have been extensively studied as a promising support or even an alternative for existing anti-obesity therapies. This review is dealing with the anti-obesity potential of edible and ethnomedicinal rhubarb species and emerging possible role of the rhubarb-derived extracts or individual compounds in the prevention of obesity and perspectives for their use in an anti-obesity treatment. A special emphasis is put on the most popular edible specimens, i.e., *Rheum rhabarbarum* L. (garden rhubarb) and *Rheum rhaponticum* L. (rhapontic rhubarb, Siberian rhubarb); however, the anti-obesity potential of other rhubarb species (e.g., *R. officinale*, *R. palmatum*, and *R. emodi*) is presented as well. The significance of rhubarb-derived extracts and low-molecular specialized rhubarb metabolites of diversified chemical background, e.g., anthraquinones and stilbenes, as potential modulators of human metabolism is highlighted, including the context of cardiovascular disease prevention. The available reports present multiple encouraging rhubarb properties starting from the anti-lipidemic action of rhubarb fibre or its use as purgative medicines, through various actions of rhubarb-derived extracts and their individual compounds: inhibition of enzymes of cholesterol and lipid metabolism, targeting of key molecular regulators of adipogenesis, regulators of cell energy metabolism, the ability to inhibit pro-inflammatory signalling pathways and to regulate glucose and lipid homeostasis contributing to overall in vivo and clinical anti-obesity effects.

## 1. Introduction

**Figure 1:**
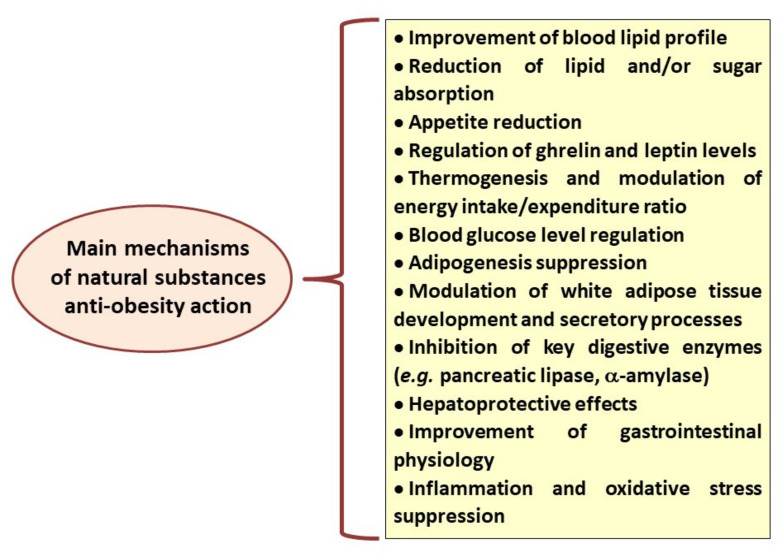
Main mechanisms of anti-obesity action recognised for different natural, plant-derived substances.

According to data reported by the World Health Organization, the number of obese subjects has nearly tripled since 1975, leading to more than 1.9 billion overweight adults in 2016 [1]. Obesity has become the largest global chronic health problem in Western civilization, and the available anti-obesity therapies are limited by many side effects, risks associated with surgical interventions and obesity relapse [2]. For that reason, lifestyle factors such as balanced diet and physical activity remain fundamental both at the stage of obesity prevention and treatment. In addition, many studies suggest that some of the plant-derived substances and herbal medicines can be helpful anti-obesity agents [3]. Their beneficial properties have been attributed to various mechanisms of action, including appetite reduction, stimulation of thermogenesis, pancreatic lipase inhibitory effects, reduction in dietary fat absorption, stimulation of lipolysis and reduction in lipogenesis (Figure 1) [4,5].

The present work covers a review of the available literature related to anti-lipidemic properties of the rhubarb-derived extracts and individual substances, use of rhubarbs in the prevention of obesity and prospects for their application in an anti-obesity treatment. A special emphasis is put on bioactive properties of two edible specimens, i.e., *Rheum rhabarbarum* L./syn. *Rheum undulatum* L. (garden rhubarb) and *Rheum rhaponticum* L. (rhapontic rhubarb, Siberian rhubarb) (www.theplantlist.org, accessed on 11 April 2022; www.worldfloraonline.org, accessed on 11 April 2022) as sources of dietary fibre and numerous bioactive (poly)phenolic compounds. However, biological properties of other rhubarb species are also presented and discussed in the context of their anti-lipidemic effects, body weight control and obesity prevention.

The review is based on in vitro and in vivo data (from both animal and human studies), originating from peer-reviewed journals indexed in international databases (i.e., Medline/Pubmed, Scopus, Science Direct/Elsevier and Springer Link/ICM) and published up to April 2022. In regard to the type of experimental design, the inclusion criteria covered conclusive studies with appropriate controls and methodology, with a special emphasis on randomized controlled trials (RCTs). To present general topics or to extend readers’ knowledge of particular aspects, well-written, comprehensive reviews were also included into this work. Editorials, conference communications, commentaries, papers from journals non-indexed in scientific databases as well as articles written in languages other than English were excluded. The main search criteria covered a combination of the “rheum” or “rhubarb” words with “obesity”, “obese” “hypecholesterolemia”, “adipocyte”, “adipogenesis” and “fibre”.

## 2. Rhubarbs as Ethnomedicinal Plants

The *Rheum* genus (rhubarb, *Polygonaceae*) includes over 60 species, commonly known as edible and medicinal plants in Asia, Europe and other regions of the world. Petioles of garden (*R. rhabarbarum/R. undulatum*) and rhapontic (*R. rhaponticum*) rhubarbs are primarily used as foods, while their underground parts provide herbal material. The current knowledge of phytochemical profile, ethnomedicinal relevance and main directions of physiological activity of individual compounds and extracts originated from *R. rhaponticum* and *R. rhabarbarum* and other rhubarbs have been extensively reviewed elsewhere [6,7,8,9]. Hence, in this paper, these issues have been only briefly mentioned. In traditional medicine, both species are used to alleviate various gastrointestinal disorders. Furthermore, mixtures based on *R. rhaponticum* were also used to cure heart problems, pulmonary system dysfunctions, and disorders related to the reproductive system, including uterine and breast pains. *R. rhabarbarum* was administered as a purgative and sedative remedy [9]. *R. officinale* Baill. (Chinese rhubarb) and *R. palmatum* L. are well-known medicinal plants widely used in traditional Chinese medicine and in many other regions of the world to treat gastrointestinal disorders, chronic renal failure inflammations and liver diseases [10]. Additionally, *Rheum emodi* Wall ex. Meissn has been known in Chinese and Ayurveda medicine, useful in the treatment of different types of cancer, ulcers, headaches, diarrhoea and liver disorders [11]. In Turkish folk medicine, *R. officinale* and *R. ribes* L. (Syrian rhubarb) are traditionally used for the treatment of obesity [12].

## 3. Rhubarb-Based Preparations in a Contemporary Pharmaceutical and Food Market

*R. officinale* (Chinese rhubarb), *R. palmatum* and other anthraquinone-rich rhubarb species, belonging to typical medicinal rhubarb specimens, are predominantly administered as laxatives [13]. They are commercially available as *Radix Rhei* or as components of different herbal mixtures, dietary supplements and tea dedicated to alleviating gastrointestinal disorders and to treat constipations.

Petioles of edible rhubarbs such as *R. rhaponticum* and *R. rhabarbarum* are used for culinary purposes, while their underground parts are a source of herbal material. The estrogenic activity of *R. rhaponticum* has been well-documented in animal and clinical studies. It has been primarily attributed to the presence of different hydroxystilbenes and their derivatives, including rhaponticin, desoxyrhaponticin, rhapontigenin, desoxrhapontigenin, resveratrol and piceatannol. A special extract (ERr 731^®^) prepared from roots of this plant specimen has been registered as a bioactive component of herbal preparation recommended to alleviate menopausal symptoms, as an alternative to the conventional hormone replacement therapy [14]. The stilbene-containing *R. rhaponticum* extracts are also available in different dietary supplements. In addition, the contemporary food and pharmaceutical market offers numerous dietary supplements based on powdered rhubarb rhizome and fibre. Components of *R. rhabarbarum* were also reported to bind to estrogen receptors ERα and ERβ [15], but the hormone-like activity of this species has been hitherto demonstrated using only in vitro experimental models.

**Figure 2:**
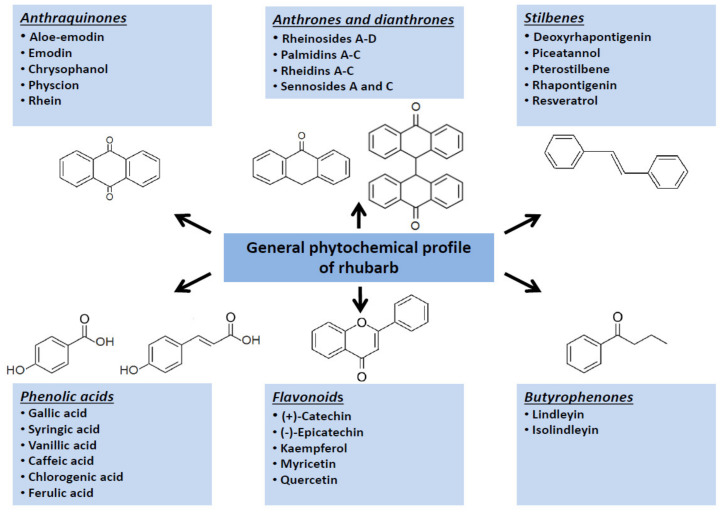
Graphical summary of main groups of the specialized metabolites synthesized by rhubarb species.

Owing to a protective activity of phytoestrogens on homeostasis [16,17], this activity of rhubarb-extracts, in some extent, may be helpful in maintaining of proper body weight and reduction in incidence and severity of cardiovascular diseases. However, in the context of a direct impact on development and progression of obesity, other molecular mechanisms as well as other bioactive phytochemicals seem to be more crucial. Originally, scientific attention was focused on the rhubarb fibre. Further studies (presented below) extended this approach by findings confirming that low-molecular bioactive metabolites from rhubarbs might affect different metabolic pathways related to obesity development, including the endocrine activity of adipose tissue, lipid metabolism and glucose uptake. Main groups of the low-molecular specialized metabolites synthesized by various rhubarb species are shown in the (Figure 2).

## 4. Hypolipidemic Effects of Rhubarb: Not Only the Fibre

Edible varieties of rhubarb are considered a rich source of various types of dietary fibre. Comparative analyses of the fibre content in vegetables and fruits revealed that rhubarb stalks contained 26.7% of insoluble fibre, whereas 54.9% were residues after digestion with Driselase. In apple, insoluble fibre amounted to 9.8%, and digestion residue content was 46.2%. For carrot, the comparative values were 10.3% and 34.6%, respectively [18]. The total dietary fibre in a preparation from fresh and frozen rhubarb stalks amounted to 741 g/kg of dry weight (d.w.), including 659 g of insoluble dietary fibre/kg d.w. and 82 g of soluble dietary fibre/kg d.w. [19]. It has been also reported that 140 g of stewed rhubarb provided 3.8 g of the total NSP (non-starch polysaccharides), 2.2 g of insoluble NCP (non-cellulosic polysaccharides) and 0.7 g of soluble NCP fibre [20].

Although the hypolipidemic action of dietary fibre has been well established [21], the use of *Rheum* species as a source of natural fibre or other lipid-lowering substances is still under examination. Current knowledge of physiological effects of rhubarb-derived fibre is mainly based on results originated from experiments employing animal models of hypercholesterolemia and obesity; however, data from studies on human subjects are also available (for details see Table 1). The *R. rhaponticum* fibre has been shown to effectively decrease plasma cholesterol in animals fed a high-cholesterol diet, although its effect on cholesterol synthesis in hepatocytes was not found [22]. As evidenced, the fibre may influence secretory processes in the liver and gallbladder functions by both direct and indirect mechanisms. Directly, it binds bile acids [23]. The binding rate of taurocholic acid (TCA) for rhubarb fibre was established to be 6.2 µmol/100 mg and increased linearly with the fibre concentration. For a comparison, the TCA-binging rate for cereal bran ranged between 2.3 and 3.5 µmol/100 mg, and for cellulose, this rate amounted to 1.0 µmol TCA/100 mg [24]. The bile-binding rate for the different digested cereal products and alcohol-insoluble substances from fruits and vegetables such as apples, strawberries, rowan berries, carrots, white cabbage, red beets and sugar beet pulp attained 1.21–1.77 µmol bile acids/100 mg of the examined preparation [25].

Indirectly, rhubarb fibre regulates cholesterol metabolism. In vivo examination revealed a stimulatory effect of rhubarb fibre on the expression of cholesterol 7α-hydroxylase gene and excretion of bile acid in cholesterol-fed C57BL/6J mice [19]. Furthermore, the hypocholesterolemic effect of rhubarb fibre was also observed in hypercholesterolemic men, consuming 27 g of rhubarb stalk fibre daily for 4 weeks [23].

Contrary to the above results, a study on diabetic rats maintained on a diet rich in rhubarb fibre, i.e., 50 g of rhubarb stalk fibre/kg of body weight (b.w.), did not demonstrate a beneficial effect on the plasma cholesterol or triacylglycerol concentrations in the examined animals [26]. Moreover, the literature clearly indicates that the lipid-lowering action of rhubarb may be also dependent on the presence of other constituents, i.e., low -molecular phytochemicals as well as synergistic action of different types of phytocompounds. Based on studies from the 1990s, demonstrating the cholesterol-lowering properties of pectins [27,28,29], the hypolipidemic properties of rhubarb were strictly attributed to its fibre content. Later decades revised this point of view and revealed that stilbene compounds such as resveratrol [30,31,32], rhaponticin and rhapontigenin [33,34] display anti-hyperlipidemic effects in vivo (Table 2). Administration of rhaponticin and rhapontigenin to animals fed a high-cholesterol diet significantly improved the blood lipid profile and reduced the extent of pathological changes in fatty liver [33].

Among reports dealing with the anti-obesity properties of stilbene-type compounds, the action of resveratrol has been described and evidenced in the widest extent of examinations, including clinical trials. Although the anti-obesity [35,36] effects of resveratrol appeared to be a very promising research trend, results derived from studies involving human subjects are inconclusive, or even, in some cases, research hypotheses have failed [37,38,39,40,41,42,43,44,45,46,47,48,49,50,51,52] (Table 3). The synergistic activity of resveratrol and other substances (either polyphenolics [53,54] or drugs [55]) was examined to find an effective combination for treatment of obesity or its complications. In a randomized controlled trial, study participants were assigned into one of four groups, i.e., placebo, resveratrol (100 mg), orlistat (120 mg), or a combination of orlistat-resveratrol (O-R; 120 mg + 100 mg) group. After 6 months, results obtained for the resveratrol monotherapy group did not significantly differ from the placebo group. The anti-obesity action and weight loss were found in both the orlistat and O-R groups. However, the most effective reduction in BMI, fat mass and waist circumference was found in subjects taking the O-R combination [55].

Data obtained for the stilbenoids are also supported by results from studies on other individual compounds that are present in the rhubarb profile, e.g., aloe-emodin [56] and physcion [57]. Additionally, emodin displayed a hypocholesterolemic effect in vitro and in vivo, through the capability of bile salts binding and increasing the expression of cholesterol 7-alpha-monooxygenase (CYP7A1) [58]. In obese mice, emodin (40 or 80 mg/kg b.w., for 6 weeks) modulated the adipose tissue physiology, and decreased the body weight and the blood lipid level. As was demonstrated by the increased levels of markers of beige adipocytes (i.e., Cd137, Tmem26 and Tbx1 mRNA), the browning processes were stimulated in the white adipose tissue [59].

## 5. Molecular Targets for Rhubarb-Derived Substances

### 5.1. Inhibitory Effects on Key Enzymes Related to Lipid Absorption and Metabolism

Administration of different types of anti-obesity drugs have been proven to have a wide spectrum of side effects, including headache, insomnia, nervousness, constipation, hypertension as well as an increased risk of cardiovascular complications. For example, due to the prevalence of risk of adverse effects over the benefits, the European Medicines Agency (EMA) recommended the removal of sibutramine from the pharmaceutical market in the European Union after only several years of use [60]. Among synthetic anti-obesity drugs, orlistat (tetrahydrolipstatin), an inhibitor of pancreatic lipase (EC 3.1.1.3), is the only currently approved weight loss medicine for the long-term treatment of obesity [61,62]. The molecular target of this drug is the pancreatic lipase (PL), which is essential for digestion of dietary triglycerides. For that reason, the search for new and more effective inhibitors (including natural compounds) of this hydrolytic enzyme has gained increasing attention [63]. Moreover, numerous reports demonstrated the ability of phytochemicals belonging to various classes to inhibit PL activity [64,65,66,67,68,69,70,71,72,73]. Most of the examined plant-derived substances were weaker inhibitors of the pancreatic lipase than the reference drug, but some of them, i.e., ferulic acid and kaempferol-3-*O*-rutinoside, displayed comparable PL-inhibitory efficiency. Some of these potential inhibitors of PL, e.g., phenolic acids as well as quercetin and kaempferol derivatives, are present in rhubarb species (Table 4). Synergistic interactions of different compounds in the inhibition of 3T3-L1 PL activity and preadipocyte differentiation have been also evidenced [74]. An PL inhibitor screening of the 37 plant extracts revealed that only 6 of them had moderate or strong anti-lipase activity (more than 30% of inhibition). Extracts from *Prunella vulgaris* L. and *Rheum palmatum* were identified as the two most potent inhibitors, displaying 74.7% and 53.8% of inhibitory effectiveness towards PL, respectively [75]. In another screening, a methanolic extract from *R. ribes* rhizome inhibited the PL activity by 43%. The most effective plant preparation in that study, i.e., *Quercus infectoria* G. Olivier galls, reduced the PL activity by 85% [76]. Furthermore, galloyl glucosides and galloylproanthocyanidins isolated from rhubarb (*R. palmatum*) roots (*Rhei Rhizoma*) were described as promising inhibitors (acting at micromolar concentrations) of squalene epoxidase, a key enzyme of the cholesterol biosynthesis pathway [77].

According to the literature, rhubarb contains natural inhibitors of soluble epoxide hydrolase (sEH), which is believed to be one of the most important molecular targets in the therapy of cardiovascular diseases as well as other disorders. sEH is a major enzyme responsible for the hydrolysis of epoxy-fatty acids (incl. arachidonic, linoleic, eicosapentaenoic, and docosahexaenoic acid epoxides). The plant-derived compounds or extracts have been examined as potential inhibitors of this enzyme. For example, ethanol extract of *Sophora flavescens* Aiton roots inhibited this enzyme with IC_50_ = 2.07 µg/mL [78]. sEH-inhibitory effects were also found for phenolic compounds isolated from the rhizomes and roots of *Gentiana scabra* Bunge [79], extracts from aerial parts of *Tetrastigma hemsleyanum* (King) Chantaran. and J. Parn. [80] and compounds isolated from roots of *Lycopus lucidus* Turcz. ex Benth. [81].

Both the methanol extract, n-hexane, chloroform and butanol fractions from *R. rhabarbarum* displayed sEH-inhibitory efficiency, but mechanistic analyses revealed evident diversity in actions of individual components of the tested extracts. Piceatannol 3′-*O*-β-D-glucopyranoside was found to be a competitive sEH inhibitor. In contrast, resveratrol, desoxyrhaponticin, rhaponticin, isorhapontin, desoxyrhapontigenin, along with other compounds such as astringin, chrysophanol-8-*O*-β-D-glucopyranoside and aloe emodin acted as the mixed-type inhibitors. Rhapontigenin and emodin were non-competitive inhibitors of sEH. Among the examined compounds, astringin was the most effective sEH inhibitor (IC_50_ = 2.5 ± 0.5 µM), and the weakest one was desoxyrhaponticin (IC_50_ = 53.2 ± 4.4 µM) [82]. Unfortunately, in the case of the search for the rhubarb-derived inhibitors of sEH, only preliminary in vitro results are available, although similar works on sEH inhibitors from other plants are more advanced and have reached the level of in vivo studies [83].

### 5.2. Modulation of the Adipose Tissue Physiology

**Figure 3:**
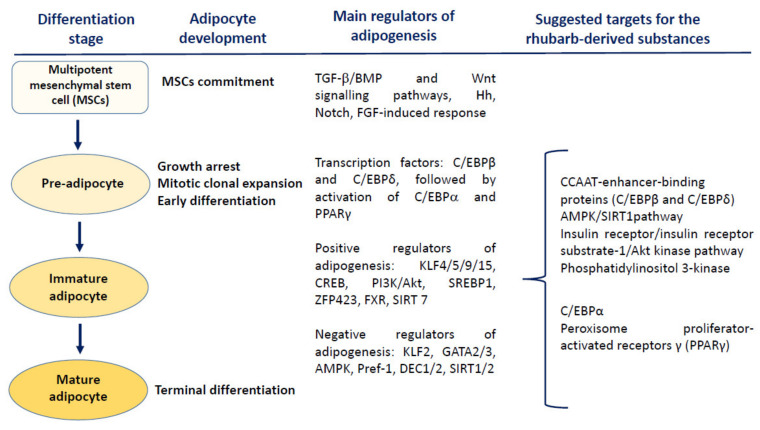
Modulatory effects of the plant-derived substances on different steps of adipogenesis. The wingless-type MMTV integration site (Wnt) signalling, the growth factor-beta (TGF-β)/bone morphogenic protein (BMP) signalling, Hedgehogs (Hh), Notch, and the fibroblast growth factor (FGF)-dependent response are the main mechanisms regulating the commitment of multipotent mesenchymal stem cells (MSCs). Early stages of adipocyte differentiation are primarily regulated by C/EBPβ and C/EBPδ, responsible for the induction of C/EBPα and PPARγ—the central positive modulators of adipogenesis. The rhubarb-originated substances may interfere with different pathways controlling the adipocyte differentiation and maturation, including the blockade of clonal expansion as well as downregulation of PPARγ and C/EBPα. Abbreviations and explanations: AMPK—AMP-activated protein kinase; KLF 2—Krüppel-like factor; Pref-1—preadipocyte factor 1; DEC1,2—transcription factors DEC1,2; GATA 2/3—GATA-binding factor 2/3; SIRT1/2—sirtuin 1,2; TGF-β—tumour growth factor beta; PI3K/Akt—phosphatidylinositol 3-kinase and Akt/Protein Kinase B pathway; KLF4/5/9/15—Krüppel-like factors 4,5,9, and 15; CREB—cAMP response element-binding protein; SIRT7—sirtuin 7; SREBP1—sterol regulatory element-binding protein 1; ZFP423—C2H2 zinc-finger protein; FXR—farnesoid X receptor; C/EBPα, C/EBPβ and C/EBPδ—CCAAT/enhancer-binding protein alpha, beta and delta; PPARγ—peroxisome proliferator-activated receptor gamma.

Although the energy storage is commonly believed to be the main role of white adipose tissue, current knowledge of human physiology evidently indicates its important endocrine activity, influence on numerous molecular pathways and modulatory effect on homeostasis [84]. Studies on anti-obesity actions of various natural substances and herbal remedies are focused on several points that may be critical in the regulation of either adipogenesis or secretory activity of the adipocytes. During this multistep process, phytochemicals (including compounds present in rhubarbs) may interact at the mitotic clonal expansion, early differentiation and terminal differentiation stages of adipogenesis (Figure 3). Due to multifactorial etiology and pathophysiology of obesity, many mechanisms underlying the anti-obesity effects of phytochemicals still remain only partly elucidated. Existing evidence indicates that the main targets for most plant-derived anti-obesity agents are key regulators of adipogenesis such as CCAAT/enhancer-binding proteins (C/EBPs) and the peroxisome proliferator-activated receptors (PPARs), as well as the AMP-activated protein kinase (AMPK), a regulator of energy metabolism [85,86,87]. An additional aspect of the beneficial action of phytochemicals in obese subjects may be their anti-inflammatory properties, including the ability to inhibit the nuclear factor kappa-light-chain-enhancer of activated B cells (NF-κB)-triggered signalling [88]. Exemplary molecular targets for phytochemicals are the phosphatidylinositol 3-kinase and Akt/Protein Kinase B (PI3K/Akt) pathway, the IκBα kinase/c-Jun N-terminal kinase (IKK/JNK), and Janus kinase/signal transducers and activators of transcription (JAK/STAT)-mediated response [89].

The literature provides very few data originating from studies on rhubarb extracts; however, some more information can be deduced from results reported by works on individual phytochemicals that are present in these plants. Inhibitory effects of natural substances on adipogenesis or metabolic activity of adipose tissue were found for compounds belonging to diverse phytochemical classes, e.g., stilbenes and anthraquinones. The newest literature emphasizes a pleiotropic activity of resveratrol, including the modulation of gene expression and affecting diverse molecular targets such as AMPK, the peroxisome proliferator-activated receptor co-activator-1α (PGC-1α) as well as sirtuin-1 (SIRT1) deacetylase [90]. The anti-adipogenic effects of another stilbene, i.e., piceatannol, seem to be mainly targeted to early phases of development of adipose tissue. The anti-adipogenic properties of piceatannol involved reduction in the mitotic clonal expansion, decrease in C/EBPβ, PPARγ, and C/EBPα mRNA levels and inhibition of the insulin receptor-dependent signalling (the insulin receptor/insulin receptor substrate-1/Akt kinase pathway) in 3T3-L1 preadipocytes. Piceatannol was an inhibitor of the insulin receptor kinase and phosphatidylinositol 3-kinase (PI3K) activity [91]. Its ability to suppress the macrophage interactions with adipocytes may be an important mechanism of alleviation of inflammatory processes in the adipose tissue [92]. Moreover, recent data suggest that piceatannol may be a more effective inhibitor of adipogenesis in human visceral adipose-derived stem cells than resveratrol [93].

Among rhubarb hydroxyanthraquinones, rhein is currently considered as the most promising anti-adipogenic and lipolysis-stimulating factor. The anti-obesity action of rhein is through different mechanisms, including activity on the transcriptional level, as an inhibitor of adipogenesis, including adipocyte differentiation. It rhein is able to downregulate the expression of adipogenesis-specific transcription factors such as PPARγ and CCAAT-enhancer-binding protein-α (C/EBPα) and their upstream regulator, i.e., CCAAT-enhancer-binding protein-β (C/EBPβ) [94]. The inhibition of PPARγ signalling was indicated as a potential mechanism of the reduction in obesity, fat mass and the size of white and brown adipocyte tissue in animals treated with rhein [95]. Another proposed mechanism of the anti-obesity action of rhein is a regulatory effect on cholesterol homeostasis, through the liver X receptors (LXRs) antagonism [96]. Recent studies on five rhubarb-derived compounds: chrysophanol, aloe emodin, emodin, physcion, and rhein suggested stronger inhibitory effects of emodin and rhein on lipid accumulation in 3T3-L1 adipocytes, compared to the remaining three hydroxyanthraquinones. Both emodin and rhein acted through mitogen-activated protein kinase (MAPK) signalling; however, their influence on adipogenesis and lipid metabolism were diverse. While rhein reduced lipid deposition by modulation of the adipogenic transcriptional factors and lipolytic enzymes, the lipid-lowering activity of emodin involved the reduction in lipogenic enzymes. The aforementioned diversity in molecular actions may result in divergent activity of both compounds in vivo. Experiments on animals demonstrated that rhein was a stronger anti-obesity agent than emodin, and reduced plasma cholesterol by 29% (versus a 14%-decrease caused by emodin) [97].

Danthron, a natural anthraquinone derivative synthesized by rhubarb, has been shown to reduce the obesity and associated metabolic fatty liver diseases (MAFLD) in animals. It has been established that this compound is able to stimulate the binding of the retinoid X receptor alpha (RXRα)/PPARα heterodimer to the promoter of the adiponectin receptor 2 (AdipoR2), resulting in the AMPKα and PPARα pathway activation [98]. The stimulation of the AMPK/SIRT1 pathway has been also suggested as the most likely mechanism of the anti-obesity activity of chrysophanol. In rats receiving a high-fat diet, this anthraquinone was found to stimulate the expression of lipolytic genes and suppress the lipogenic genes as well as to diminish the inflammatory response [99]. Anti-diabetic and anti-obesity effects in vitro and in vivo were also found for emodin. As a selective inhibitor of the 11β-hydroxysteroid dehydrogenase type 1 (11β-HSD1), emodin regulated adipogenesis and energy metabolism in 3T3-L1 adipocytes as well as ameliorated metabolic disorders in ob/ob (B6.V-Lepob/Lepob) mice [100]. In other animal study, the inhibition of proprotein convertase subtilisin/kexin type 9 was indicated as a key mechanism of the cholesterol-lowering effect of aloe-emodin [56].

### 5.3. Metabolism and Glucose Level Regulation

The ability to regulate glucose homeostasis has been demonstrated for various types of rhubarb-derived substances, i.e., mixtures extracted with the use of water or organic solvents, low-molecular isolates and the soluble fibre. Regarding the latter, it has been demonstrated that a high fibre diet might upregulate the proglucagon mRNA and secretion of both the glucagon-like peptide-1 [GLP-1(7–37)] and insulin [101]. In other work on animals, consumption of rhubarb fibre (50 g/kg of diet) influenced the ileal synthesis of proglucagon mRNA and reduced passive permeability, without affecting the glucose transport of the small intestine [102]. Furthermore, in patients with type 2 diabetes, an administration of essential oil preparation (400 mg capsules, 3 times daily, 90 capsules in total) isolated from rhubarb stem reduced the glycosylated haemoglobin and fasting blood glucose levels [103].

Another possible molecular target for rhubarb-derived substances is the protein tyrosine phosphatase 1B (PTP1B), a negative regulator of the leptin and insulin-dependent signalling pathways. Currently available works devoted to search for effective inhibitors of this enzyme reported examinations of over few hundreds of synthetic and natural compounds, including plant extracts [104,105]. Among them, one of the available medicinal plant screenings revealed that a hot water extract from *R. rhabarbarum* roots might reduce the PTP1B activity in vitro. Moreover, studies on high-fat diet-fed C57BL/6 mice and in vitro experiments demonstrated the metabolism-regulatory activity of *R. rhabarbarum* extracts and its anthraquinone compounds—chrysophanol and physcion [106]. Other authors reported the hypoglycaemic effects of *Rheum tanguticum* Maxim. ex Balf., based on the sucrase and maltase inhibitory activities of this plant metabolites such as flavan ((-)-epicatechin 3-*O*-gallate) and phenylbutanone (lindleyin) [107].

In animals, the glucose homeostasis regulatory ability was evidenced for both individual compounds and extracts isolated from different rhubarb species (Table 5). Beneficial effects of rhubarb-derived phytochemicals included the insulin-sensitizing activity reduction in the blood triglyceride and glucose [108,109,110,111,112] levels. Interestingly, some caution has been recommended in the case of *Rheum turkestanicum*, whose rhizomes are a traditional anti-diabetic medicine in Iran. An animal study revealed that this plant rhizome extract had anti-hypertriglyceridemic activity, but no hypoglycemic or hepatoprotective effects were found [113]. The combination of metformin and the anthraquinone glycoside preparation from *R. palmatum* rhizomes administered to rats (100 or 400 mg/kg b/w., for 6 weeks) ameliorated type 2 diabetes mellitus through the regulation of the gut microbiota and activation of the GLP-1/cAMP pathway, resulting in a decrease in insulin resistance [114]. A clinical trial on patients with type 2 diabetes mellitus demonstrated the reduction in fasting blood glucose level and glycosylated haemoglobin in the intervention group, treated with capsules containing rhubarb stem extract (90 capsules in total; 400 mg/capsule, three times a day) [103].

### 5.4. Anti-Obesity Action of Rhubarb Extracts in Animal Studies

The rhubarb extract (100 mg/kg b.w., administered for 8 weeks) modulated lipid metabolism (including a stimulation of adiponectin synthesis) and significantly reduced the body weight gain in the examined mice. In parallel experiments, the anthraquinones (30 µM) inhibited PTP1B activity and enhanced the insulin sensitivity in the serum-starved 3T3L1 cells [115]. In rats treated with a 690 mg/kg b.w. dose (for 6 weeks) of the *R. palmatum* extract (a concentrated decoction), established based on recommended clinical doses for humans (according to the *Pharmacopoeia of the People’s Republic of China*), a high-fat-diet-induced hepatosteatosis was significantly alleviated. The treatment significantly lowered the liver triglyceride levels, liver weight and improved glucose tolerance; however, a high dose of the extract, i.e., 1300 mg/kg b.w., was less efficient. The stimulating AMPK activity of the examined extract has been suggested as the most probable molecular mechanism of the observed effects [116].

The treatment with *R. undulatum* extracts not only reduced the low-density-lipoprotein cholesterol and increased the high-density-lipoprotein cholesterol in rats, but also mitigated vascular inflammation as confirmed by measurements of the vascular NF-κB-p65, adhesion molecules ICAM-1 and VCAM-1 as well as E-selectin levels [117]. In an animal model of atherosclerosis, the lipid-lowering and plaque-stabilizing properties associated with anti-inflammatory effects were found for aqueous extract from *R. officinale* [118]. A four-week therapy with hydroalcoholic extract from *R. ribes* L. root (150 mg/kg b.w./day) reduced the glucose, cholesterol and triglyceride levels in diabetic rats. Moreover, the reduction in low-density-lipoprotein (LDL) level achieved concentrations comparable to those recorded in the control group [119]. Likewise, the cholesterol-lowering effects were observed in rabbits having hypercholesterolemia, after a treatment (4 g/kg b.w./day, for 2 weeks) with ethanolic and aqueous extracts from *R. ribes* stalks [120]. Recent animal studies indicate that rhubarb supplementation may prevent diet-induced obesity. In C57BL6/J mice fed a high-fat and high-sucrose (HFHS) diet, enriched with 0.3% (g/g) of *R. palmatum* extract (for 8 weeks), the accumulated fat mass and weight gain were reduced, when compared to animals fed an HFHS diet, without the plant extract. Furthermore, in mice supplemented with the rhubarb extract, fat pads (visceral, epididymal and subcutaneous) were similar to those found in control animals (mice fed a control diet—D12450H; research diet). Rhubarb supplementation also resulted in the prevention of diet-induced liver steatosis as well as in increased hepatic cholesterol and inflammatory markers and beneficial effects on adipose tissue, including a trend towards a decreased infiltration by macrophages. Immunohistological analyses demonstrated that adipocyte morphology in mice supplemented with rhubarb extract was similar to adipocytes from control animals. In addition, those effects were associated with an increased amount of *Akkermansia muciniphila* in the colon [121], which is a commensal bacterium of the gut mucus, displaying beneficial effects for basal metabolism and associated with reduced risk of obesity [122].

### 5.5. Anti-Obesity Action of Rhubarb Extracts in Clinical Trials

A randomized, double-blind and placebo-controlled trial involving 83 patients with diagnosed atherosclerosis revealed that treatment with *R. officinalis* hot water extract (50 mg/kg b.w.) decreased the serum total cholesterol and the LDL cholesterol [123]. In contrast, administration of the *R. emodi* stem extract to patients with type 2 diabetes mellitus (taking a total amount of 90 extract capsules: 3 capsules/day, 400 mg of the extract/capsule) did not reveal any effect on body weight; however, a decrease in systolic and diastolic blood pressure was observed [124].

### 5.6. Laxative Effects of Rhubarb-Based Extracts and Preparations

In Western civilization, laxative abuse as a method supporting the weight loss and adverse effects of these substances constitutes a serious problem [125]. On the other hand, the laxative properties of natural compounds are an important element of the treatment of constipation associated with various diseases. The purgative effects of rhubarb are a result of the presence of different substances, mainly anthraquinones. For that reason, the anthraquinone-rich rhubarb specimens such as *R. officinale* and *R. palmatum* are often used as purgative medicines. The anthraquinone concentration in roots of these two rhubarb species typically ranges up to about 5% of dry mass [126,127]. Other purgative rhubarb components are anthrones and dianthrone compounds, including rheinosides A–D; palmidin A, B and C; rheidin A, B and C; as well as sennosides A–F [128]. In Asian countries, *R. emodi* is also traditionally recommended for purgative therapy. Interestingly, while larger doses of *R. emodi* are a natural laxative, at small doses, this plant was administered to treat dysentery and diarrhea [129].

According to the literature, the presence of anthraquinone glycosides with 1,8-dihydroxy groups and without hydroxyl groups in the 2, 3, 6 and 7 positions may be responsible for the effect of “watery diarrhea”, occurring after using of some rhubarb-based treatment [130]. Rhubarb contains both free anthraquinones (e.g., aloe-emodin, emodin, rhein, chrysophanol and physcion) and conjugated forms, containing the β-glycoside bonds. The anthraquinone conjugates are considered to be the exact medicinal components of the rhubarb-derived material. The presence of the β-type glycoside bonds in conjugates prevents their hydrolysis by α-glucosidase in the upper gastrointestinal tract and, as a consequence, they are hydrolysed to free anthraquinones by bacterial β-glucosidases in the colon. Recently, a Chinese research group designed oral colon-specific drug delivery granules, containing the total free anthraquinones to improve their purgative effect and to reduce anthraquinone nephrotoxicity [131,132]. The postprandial hyperlipidemia-lowering effect and improvement in gastrointestinal transit in diabetic rats was reported for aqueous extract from the rhizome of *R. palmatum*, administered at doses of 150 and 300 mg/kg b.w. The anthrone content in the examined rhubarb extract was not less than 0.5% [133]. A comparative study of three *R. palmatum*-containing traditional Chinese purgative medicines Ta-Cheng-Chi-Tang (TCCT), Xiao-Chen-Chi-Tang (XCCT) and Tiao-Wei-Chen-Chi-Tang (TWCCT) did not reveal their weight-lowering effects in rats fed on a high-fat diet. However, the examined extracts improved the blood serum lipid profile. In addition, both XCCT and TWCCT significantly attenuated hypercholesterolemia and the TWCCT preparation also reduced hypertriglyceridemia in rats [134].

Rhubarbs with lower content of anthraquinones (e.g., *R. rhabarbarum*) display mild laxative effects, but they also are used for production of different dietary supplements and food products dedicated to improving the intestine physiology and to prevent constipation. These products are available in a form of herbal mixtures, capsules or even candy-like bars enriched with the plant-derived fibre and low-molecular phytochemicals.

## 6. Concluding Remarks

Disrupted balance between an amount of taken calories and energy expenditure as well as complex physiological activity of both white and brown adipose tissue are essential pro-obesity factors. Different rhubarb species have been demonstrated to display a wide range of biological activities that may be relevant for reduction in cardiovascular risks, including improvement in glucose and lipid metabolism. The original findings on the health-promoting action of the rhubarb-derived substances were typically focused on the cholesterol-lowering role of the fibre. Currently, it is known that also other, low-molecular phytochemicals may be important for the cardioprotective, lipid-lowering and anti-adipogenic effects of these plants. However, the pharmacological significance of the rhubarb-based preparations in a context of the anti-obesity action still remains only partly recognized.

Literature evidence has indicated that the rhubarb-derived compounds and extracts may act at different molecular and physiological levels. The pathophysiology of obesity is multifactorial and includes many undesirable changes, such as alterations in blood lipid profile, enhanced adipogenesis, increased body fat mass, hyperglycemia, chronic inflammation in adipose tissue, oxidative stress, and others. Results from different types of examinations have demonstrated that rhubarb-derived substances have some potential to reduce cardiovascular risks. On the other hand, it should be noted that many works devoted to anti-obesity action of rhubarb-derived substances have stuck at a level of in vitro tests; thus, a reliable evaluation of their physiological and pharmacological effects is not possible yet. Moreover, findings from studies on individual phytochemicals (e.g., works on the anti-obesity properties of resveratrol) cannot be directly extrapolated to the effects of rhubarb preparations containing these phytochemicals. In addition, there is negligible evidence of using rhubarb extracts combined with other natural or synthetic substances (including drugs). The synergistic action of different substances seems to be a very promising research trend, and this approach could give better results. For example, a reduction in body weight gain was observed in an animal study on different combinations of extracts originating from other plants (i.e., preparations from leaves from *Phyllostachys pubescens* J. Houz. and roots of *Scutellaria baicalensis* Georgi) [135].

Furthermore, most in vivo studies have been conducted using animal models of obesity. The available data from clinical studies provide only a fragmentary insight into the pharmacological potential of these plants. Thus, the above-presented results point to the need for further and more extensive studies using standardized rhubarb extracts/preparations aimed at establishing their exact anti-obesity relevance in vivo and in humans.

## Figures and Tables

**Table 1:** Hypolipidemic action of the rhubarb-derived fibre.

The Examined Substances	Type of Study	Experimental Model, Doses and Concentrations	Main Effects of the Rhubarb Fibre and Stilbenes Administration	References
**ANIMAL STUDIES**				
*R. rhaponticum* stalk-derived preparation containing 74% of dietary fibre/dry mass (incl. 66% insoluble and 8% soluble)	animal	mice fed with cholesterol-enriched diet with 5% of rhubarb stalk fibre, for 4 weeks	↓ the acyl CoA: cholesterol acyltransferase (ACAT) activity; no effects on the cholesterol-rich diet enhancement of the β-hydroxyβ-methyl coenzyme A reductase (HMGR) activity	[22]
*R. rhaponticum* stalk fibre	animal	cholesterol-fed C57BL/6J mice receiving the fibre-rich diet (50 g/kg b.w.) for 4 weeks	↓ plasma cholesterol (−13%); ↓ the hepatic concentrations of total cholesterol (by 34%) and cholesteryl esters (by 34%); ↓ acyl CoA: cholesterol acyltransferase activity; ↓ the faecal bile acid loss; ↓the gallbladder bile acid pool	[19]
*R. rhaponticum* stalk fibre	animal	the diabetes-prone and the streptozotocin-induced diabetic rats receiving the fibre-rich diet (50 g/kg b.w.) for 2 weeks	No effect on the plasma cholesterol and triacylglycerol levels in diabetic rats	[26]
**HUMAN STUDIES**				
the rhubarb-stalk-derived preparation containing 74% dietary fibre/dry mass (incl. 66% insoluble and 8% soluble)	human	Ten hypercholesterolemic men (BMI of 27.9 ± 3.8 kg/m^2^); 27 g of rhubarb fibre/day, for 4 weeks	↓ serum total cholesterol (−8%) and LDL cholesterol (−9%); no changes in HDL cholesterol level; a return of the cholesterol-lowering effect to baseline after the fibre supplementation withdrawal for one month	[23]

**Table 2:** Exemplary data originated from animal studies on the hypolipidemic and anti-obesity properties of stilbenes that are present in rhubarb.

The Examined Substances	Experimental Model, Doses and Concentrations	Main Effects of the Stilbene Administration	References
Rhaponticin and rhapontigenin isolated from *R. rhabarbarum* roots	rats fed a high-cholesterol diet, followed by oral rhapontin or rhapontigenin treatment (1, 2.5 and 5 mg/kg b.w. (body weight)/day)	↓ the serum lipid level; ↑ HDL cholesterol; improvement in the degenerating fatty liver structure; the aspartate aminotransferase (AST) and the alanine aminotransferase (ALT) levels comparable to the control group	[33]
Rhaponticin from *R. rhabarbarum* roots	KK/Ay type 2 diabetic mice treated with rhaponticin (125 mg/kg b.w., 4 weeks)	↓ the plasma triglyceride, LDL, cholesterol, non-esterified free fatty acids; ↓ lactate dehydrogenase, creatine kinase, AST and ALT activities	[34]
Resveratrol	high-fat diet (HFD)-fed C57BL/6 J mice, a daily dose of 200 mg/kg b.w. of resveratrol, for 8 weeks	anti-hypercholesterolemic effects: improvement in serum lipid parameters, ↓ hepatic cholesterol, ↓ body weight, ↑ bile acid pool size, ↑ liver CYP7A1 mRNA expression and CYP7A1 enzyme activity	[30]
	apoE-deficient mice fed an atherogenic diet containing 0.02% resveratrol (*w/w*), for 12 weeks	↓ the plasma total cholesterol, LDL cholesterol, non-high-density-lipoprotein cholesterol, apoB/apoA1 ratio, hepatic cholesterol and triglyceride; ↑ the plasma HDL cholesterol	[31]
	mice fed standard diet plus resveratrol (4 g/kg of food to provide a 400-mpk dose), for 8 weeks	↑ brown adipose tissue thermogenesis; ↑ mRNA of thermogenesis-related genes, incl. uncoupling protein 1 (UCP1), sirtuin 1 (SIRT1), phosphatase and tensin homolog (PTEN) and bone morphogenetic protein 7 (BMP-7) expression; ↓ fat accumulation in adipose tissue; ↓ total cholesterol and glucose levels in plasma	[35]
	C57BL/6 mice fed a high-fat diet with a low dose of resveratrol, i.e., 200 mg/kg b.w./day (HFD-RES/L) or with a high dose of resveratrol, i.e., 400 mg/kg b.w./day (HFD-RES/H)	↓ insulin resistance; ↑ expressions of pAkt, glucose transporter type 4 (GLUT4) and insulin receptor substrate 1 (IRS-1) in white adipose tissue (WAT); ↓ proinflammatory cytokine levels in serum; ↓ macrophage infiltration and C-C chemokine receptor type 2 (CCR2) chemokine expression in white adipose tissue (WAT)	[36]
	rats with hyperlipidemia; a daily dose of 20 mg/kg b.w., of resveratrol, for 30 days	↓ LDL and triglyceride levels; ↑ HDL levels in animals;	[32]

**Table 3:** Evaluation of hypolipidemic effects and anti-obesity action of resveratrol in clinical trials.

Number of Participants (*n*), Resveratrol Doses and Study Duration	Participant Diagnosis	Main Effects of Resveratrol Supplementation in the Context of an Anti-Obesity Action	References
*n* = 11; 150 mg/day, for 30 days; a randomized, placebo-controlled, double-blind and cross-over study	obesity	Calorie-restriction-like effects; reduction in the sleeping and resting metabolic rate; ↓ intrahepatic lipid content, circulating glucose and triglycerides; ↓ inflammation markers; ↓ the systolic blood pressure; improvement in the HOMA index	[37]
*n* = 24; 500 mg, 3 times/day, for 4 weeks; a randomized, placebo-controlled study	obesity (BMI > 30 kg/m^2^)	No effect on the total cholesterol, HDL, LDL, plasma triglyceride and blood pressure; no changes in the resting metabolic rate and lipid oxidation	[38]
*n* = 28; 75 mg/day, for 6 weeks; a randomized, placebo-controlled, double-blind, cross-over study	obesity (BMI of 33.3 ± 0.6 kg/m^2^)	No effects on blood pressure; the flow-mediated dilatation (FMD) increased by 23%	[39]
*n* = 45; 150 mg/day, for 4 weeks; a randomized, placebo-controlled, cross-over study	overweight or obesity (BMI of 25–35 kg/m^2^)	No effects on metabolic risk markers related to cardiovascular health	[40]
*n* = 50; 500 mg/day, for 12 weeks; a randomized, placebo-controlled, double-blind study	overweight (BMI of 28.35 ± 3.49 and 28.75 ± 3.50 kg/m^2^, for the intervention and placebo group, respectively); non-alcoholic fatty liver disease	Reduction in BMI, waist circumference, HDL cholesterol and apo A1 both in intervention and placebo group; no differences in the above parameters between these groups; ↓ alanine transferase (ALT) and hepatic steatosis, compared to placebo	[41]
*n* = 8; 1000 mg once a day for a week, then 2000 mg/day for the next week; a randomized, placebo-controlled study	overweight or obesity (BMI of 27.0–40.0 kg/m^2^), mild hypertriglyceridemia	No effects on insulin sensitivity and blood plasma triglyceride level; ↓ apoB-48 and apoB-100 production rate	[42]
*n* = 10; 150 mg/a day, for 30 days; a randomized, placebo-controlled, double-blind, cross-over study	obesity (BMI of 32 ± 1 kg/m^2^)	Suppression of postprandial glucagon responses; no changes in fasting glucagon levels	[43]
*n* = 32; 300 mg/day or 1000 mg/day for 90 days; a randomized, placebo controlled, double-blind study	overweight or obesity (BMI of 25.0–34.9 kg/m^2^)	↓ glucose levels compared to placebo; no changes in blood pressure, body weight and waist circumference	[47]
*n* = 11; 150 mg/day for 30 days; a randomized, placebo-controlled, double-blind, cross-over study	obesity (BMI of 28–36 kg/m^2^)	↓ adipocyte size; changes in the adipose tissue morphology: reduction in the proportion of large and very large adipocytes; increase in small adipocytes; enhanced adipogenesis	[44]
*n* = 23; 200 mg/day for 26 weeks, a placebo-controlled study	overweight (BMI of 25–30 kg/m^2^), healthy older adults (50–80 years)	↓ body fat and leptin increase compared to placebo; no significant changes in body weight, BMI or blood pressure compared to placebo	[45]
*n* = 161; 100 mg of resveratrol or 120 mg orlistat + 100 mg resveratrol (O-R group), 3 times a day, for 6 months; the participants consumed 500 k calories fewer than the usual diet; a randomized, placebo-controlled study	obesity (BMI of 30.0–39.9 kg/m^2^)	No significant changes in the group treated with resveratrol solely; ↓ BMI, waist circumference and fat mass in the orlistat-treated and O-R groups; the most effective one was the O-R combination	[55]
*n* = 74; 150 mg or 1000 mg/day, for 16 weeks; a randomized, placebo-controlled study	obesity (BMI of 33.8 ± 0.44 kg/m^2^)	No effect on blood pressure, body composition, lipid deposition in the liver or striated muscle; no beneficial effect on glucose and lipid metabolism; 1000 mg dose increased the total cholesterol and LDL compared to placebo group	[46]
*n* = 45; 75 mg twice a day, for 4 weeks; a randomized, placebo-controlled study	overweight or slight obesity (BMI of 28.3 ± 3.2 kg/m^2^)	No changes in plasma biomarkers of endothelial function or inflammation (both in the fasting state and postprandial phase); no changes in serum triglyceride and insulin level	[49]
*n* = 38; a combination of 282 or 80 mg/day of the epigallocatechin-3-gallate and resveratrol (EGCG + RES), for 12 weeks; a randomized, placebo-controlled study	overweight or obesity (BMI of 29.7 ± 0.5 kg/m^2^)	↓ visceral adipose tissue mass; no effect on insulin-stimulated glucose disposal, endogenous glucose production or lipolysis;	[53]
*n* = 25; 282 or 80 mg/day of the EGCG + RES, for 12 weeks; a randomized, placebo-controlled study	overweight or obesity (BMI of 29.7 ± 1.1 kg/m^2^)	No changes in adipocyte size or surface area in abdominal subcutaneous adipose tissue; EGCG + RES downregulated pathways contributing to adipogenesis, cell cycle and apoptosis in the abdominal subcutaneous adipose tissue	[54]
*n* = 112; 75 mg, twice a day, for 12 weeks; a randomized, placebo-controlled study	overweight or obesity (BMI ≥ 27 kg/m^2^), insulin resistance	No effects on cardiometabolic risk parameters and liver fat content	[48]
*n* = 28; 1000 mg, twice a day, for 30 days; a randomized, placebo-controlled study	obesity (BMI of 30–40 kg/m^2^), metabolic syndrome	No changes in insulin resistance; no changes in adipose tissue metabolism	[50]
*n* = 41; 150 mg/day, for 6 months; a randomized, placebo-controlled study	overweight or obesity (BMI of 27–35 kg/m^2^)	No effects on intrahepatic lipid level, energy metabolism, blood pressure, physical performance, quality of life and sleep	[51]
*n* = 25; 250 mg/day, with physical training and diet, for 3 months; a randomized, placebo controlled, double-blind study	obesity (BMI ≥ 30 kg/m^2^), metabolic syndrome	Resveratrol potentiated beneficial effects of diet and physical training; ↓ VLDL and the total cholesterol in blood plasma	[52]

BMI—body mass index.

**Table 4:** Pancreatic lipase inhibitory effects of exemplary phytochemicals, present also in rhubarbs.

Compound	Phytochemical Classification	Pancreatic Lipase Inhibitory Effects		References
		IC_50_ for the Examined Compound	IC_50_ for Orlistat	
Caffeic acid	Phenolic acids	401.5 μM	4.0 μM	[64]
Chlorogenic acid		110.0 μM 114.0 μM	0.23 μM ND	[65]
*p*-Coumaric acid		170.2 μM	4.0 μM	[64]
Ellagic acid		44.78 μM	0.23 μM	[65]
Ferulic acid		2.49 μM	4.0 μM	[65]
Cyanidin-3-rutinoside	Anthocyanidins and their derivatives	188.28 μM	ND	[67]
		59.4 μM	31.7 μM	[68]
Delphinidin-3-glucoside		223.26 μM	ND	[68]
Procyanidin B2	Proanthocyanidins	7.96 μM	ND	[69]
*cis*-Piceid	Stilbene derivatives	76.1 μM	0.7 μM	[70]
*trans*-Piceid		121.5 μM	0.7 μM	[70]
*trans*-Resveratrol		>200 μM	0.7 μM	[70]
Kaempferol-3-*O*-rutinoside	Flavonoids and their glycosides	2.9 μM	1.45 μM	[71]
Quercetin		421.1 μM	ND	[72]
		146 μM	1.45 μM	[71]
Quercetin-3-*O*-β-D-glucuronide		94 μM	ND	[73]
Rutin		149 μM	1.45 μM	[71]

ND—not determined.

**Table 5:** The ability of rhubarb-derived substances to regulate the glucose homeostasis demonstrated in animal studies.

The Examined Rhubarb Compounds or Extracts	Experimental Model/Doses	Main Findings	References
desoxyrhapontigenin, emodin and chrysophanol, from roots of *R. rhabarbarum*	mice	↓ postprandial hyperglycaemia by 35.8, 29.5, 42.3%, respectively	[109]
70% ethanol *Rhei Rhizoma* extract	streptozotocin-induced diabetes in mice/5 mg/kg b.w. (body weight), 8 weeks	↑ insulin-stimulated glucose uptake, ↓ carbohydrate digestion via inhibiting alpha-glucoamylase	[109]
decoction from *R. turkestanicum* rhizome	diabetic rats/200–600 mg/kg b.w, 3 weeks	no effects on serum glucose, ↓ serum triglyceride level	[113]
standardized extract from *R. turkestanicum* roots	streptozotocin-induced diabetes in rats/100, 200 and 300 mg/kg b.w., 4 weeks	↓ blood glucose, ↓ diabetic changes in kidneys, liver and heart	[110]
*R. emodi* extract	rats treated with glucocorticoids/10, 20 and 30 g of rhubarb powder/kg of diet, 8 weeks	↓ blood glucose and immunity markers	[111]
anthraquinone-glycoside preparation from *R. palmatum*	high-fat diet-induced type 2 diabetes mellitus in rats/100, 200, and 400 mg/kg b.w., 6 weeks	↓ fasting blood glucose, ↓ total cholesterol and triglyceride levels, improvement in pathological changes in the liver, kidney and pancreatic tissues	[112]
